# Human gene and microbial analyses in rectal cancer complete responses to radiotherapy

**DOI:** 10.1093/bjsopen/zrad035

**Published:** 2023-05-10

**Authors:** Arielle Kae Sulit, Kasmira Wilson, John Pearson, Olin K Silander, Shienny Sampurno, Michael Michael, Robert Ramsay, Alexander Heriot, Frank Frizelle, Rachel Violet Purcell

**Affiliations:** School of Natural Sciences, Massey University, Auckland, New Zealand; Department of Surgery, University of Otago, Christchurch, New Zealand; Department of Surgical Oncology, Peter MacCallum Cancer Centre, Melbourne, Victoria, Australia; Differentiation and Transcription Laboratory, Sir Peter MacCallum Cancer Centre, Melbourne, Victoria, Australia; Sir Peter MacCallum Department of Oncology, University of Melbourne, Melbourne, Victoria, Australia; Biostatistics and Computational Biology Unit, University of Otago, Christchurch, New Zealand; School of Natural Sciences, Massey University, Auckland, New Zealand; Differentiation and Transcription Laboratory, Sir Peter MacCallum Cancer Centre, Melbourne, Victoria, Australia; Sir Peter MacCallum Department of Oncology, University of Melbourne, Melbourne, Victoria, Australia; Department of Medical Oncology, Peter MacCallum Cancer Centre, Melbourne, Victoria, Australia; Department of Surgical Oncology, Peter MacCallum Cancer Centre, Melbourne, Victoria, Australia; Differentiation and Transcription Laboratory, Sir Peter MacCallum Cancer Centre, Melbourne, Victoria, Australia; Department of Surgical Oncology, Peter MacCallum Cancer Centre, Melbourne, Victoria, Australia; Sir Peter MacCallum Department of Oncology, University of Melbourne, Melbourne, Victoria, Australia; Department of Surgery, University of Otago, Christchurch, New Zealand; Department of Surgery, University of Otago, Christchurch, New Zealand

## Abstract

**Background:**

The gold standard treatment for locally advanced rectal cancer is total mesorectal excision after preoperative chemoradiotherapy. Response to chemoradiotherapy varies, with some patients completely responding to the treatment and some failing to respond at all. Identifying biomarkers of response to chemoradiotherapy could allow patients to avoid unnecessary treatment-associated morbidity rate. While previous studies have attempted to identify such biomarkers, none have reached clinical utility, which may be due to heterogeneity of the cancer. In this study, potential human gene and microbial biomarkers were explored in a cohort of rectal cancer patients who underwent chemoradiotherapy.

**Methods:**

RNA sequencing was carried out on matched tumour and adjacent normal rectum biopsies from patients with rectal cancer with varying chemoradiotherapy responses treated between 2016 and 2019 at two institutions. Enriched genes and microbes from tumours of complete responders were compared with those from tumours of others with lesser response.

**Results:**

In 39 patients analysed, enriched gene sets in complete responders indicate involvement of immune responses, including immunoglobulin production, B cell activation and response to bacteria (adjusted *P* values <0.050). Bacteria such as *Ruminococcaceae bacterium* and *Bacteroides thetaiotaomicron* were documented to be abundant in tumours of complete responders compared with all other patients (adjusted *P* value <0.100).

**Conclusion:**

These results identify potential genetic and microbial biomarkers of response to chemoradiotherapy in rectal cancer, as well as suggesting a potential mechanism of complete response to chemoradiotherapy that may benefit further testing in the laboratory.

## Teaser

Rectal cancer patients respond differently to presurgical radiotherapy, such that biomarkers indicating efficacy of the treatment are vital to stratify patients to the most effective treatment regimen. Currently identified biomarkers have no clinical utility, possibly due to heterogeneity of the disease in patients. In this article we present a method that mitigates interpersonal variation between patients, allowing the identification of immune responses and microbes, and their possible contributions to the mechanisms of complete response to radiotherapy in rectal cancer.

## Introduction

Locally advanced rectal cancer often requires neoadjuvant chemoradiotherapy (nCRT), aiming to downstage tumours and reduce rates of local recurrence, improving survival^1,2^. The response to nCRT varies: up to 20 per cent of patients achieve a pathological complete response (pCR), up to 60 per cent demonstrate partial response, and the remainder display resistance to nCRT^1,3^. These response rates could further be increased by total neoadjuvant therapy^3^.

A pCR is defined as the absence of residual viable tumour cells in the resected specimen and the reliance on pathological confirmation of a complete response fails to identify patients that may benefit from organ preservation. Habr-Gama pioneered the adoption of a clinical complete response (cCR) as a surrogate for pCR^4^. Patients who achieve a cCR may subsequently be managed using a ‘Watch and Wait’ approach, thereby avoiding the morbidity rate associated with major resectional surgery. Although up to 25 per cent of these patients show tumour regrowth by 2 years, the majority are amenable to salvage procedures^5^.

Even though extensive work has been carried out to identify clinical and biological markers of response to radiotherapy in rectal cancer^1,6–8^, no reliable biomarkers have been validated for clinical use. A robust biomarker that selects patients likely to achieve a pCR to nCRT would allow the accurate identification of responders and increase confidence in selecting patients amenable to non-operative management. Chemoradiotherapy (CRT) is associated with significant localized and systemic side-effects and has been demonstrated to negatively impact quality of life^9^. The identification of patients unlikely to benefit from conventional CRT would result in a reduction in CRT-associated side-effects, and targeting of patients for novel treatment strategies.

Due to the largely sporadic nature of colon and rectal cancer, environmental factors are likely to play a critical role in the development of the disease, and recent international data points to the importance of the microbiome in its development and progression^10–12^. Recent reports have also demonstrated that systemic effects of the gut microbiome may contribute to treatment response in other cancer types^13,14^ and could be predictors of a favourable response to immunotherapy. In addition, gut microbiota have been shown to locally influence the treatment efficacy of irinotecan for colorectal cancer (CRC)^15^. However, although some studies on the protective effect of the microbiome on radiotherapy-induced toxicity have been carried out^16,17^, very little is known about whether or how the gut microbiome may regulate the response of the tumour to radiotherapy.

This research aimed to analyse gene expression data from a unique cohort of pre-nCRT rectal cancer tumours and their matched normal mucosa samples to identify tumour and microbial genes and molecular pathways associated with response.

## Methods

### Patients

Two prospective, consecutively sampled cohorts of patients with rectal cancer treated at Christchurch Hospital, New Zealand (study period: 2018), and at the Peter MacCallum Cancer Centre, Melbourne, Australia (study period: August 2016 to February 2019) were analysed. Patients were selected for nCRT at multidisciplinary team meetings at their respective institutions. Pretreatment biopsies of tumour tissue and adjacent, macroscopic normal tissue (>10 cm from tumour) were taken at colonoscopy, before nCRT treatment. Patients who had received previous chemotherapy or radiation therapy for treatment of their rectal tumour were excluded from the study. Patient data, including clinical staging, treatment schemes, histology and follow-up (recurrence and metastases until March 2022), was collected. Response to long-course CRT (LCCRT) was assessed histologically from surgical resection specimens, and reported using Dworak grading (Christchurch cohort) or the American Joint Committee on Cancer (AJCC) grading (Melbourne cohort). Response groups were designated as complete responders (Dworak 4/AJCC 0), near-complete responders (Dworak 3/AJCC 1), incomplete responders (Dworak 2/AJCC 2) and non-responders (Dworak 1/AJCC 3). In addition, patients who developed progressive disease or died of disease, during the course of therapy, were also designated non-responders.

The study was undertaken with ethical approval from the Health and Disability Ethics Committee of New Zealand (ethics approval number: 18/STH/40/AM01) and the Human Research Ethics Committees of Australia (ethics approval number: HREC 14/85). All participants provided written, informed consent before enrolment.

### Outcomes of interest

The primary outcome of interest was to compare human gene expression and microbial taxa abundances in different groups of responders. To address this objective, complete responders were compared with all other responders grouped together, and secondly, non-responders were compared with all other response groups. In addition, the correlation between differentially expressed genes (DEGs) and differentially abundant bacteria was investigated, as well as microbial diversity.

### RNA extraction, sequencing and processing

Tumour and normal tissue biopsies were taken at colonoscopy and immediately frozen in liquid nitrogen and stored at −80°C. RNA extraction was carried out as detailed previously^18^. Briefly, RNA was extracted from <20 mg of tissue using the RNEasy Plus Mini Kit (Qiagen, Hilden, Germany), including DNAse treatment, following tissue disruption using a Retsch Mixer Mill. Purified RNA was quantified using a NanoDrop 2000c spectrophotometer (Thermo Scientific, Asheville, NC, USA), and stored at −80°C. RNA sequencing was performed using an Illumina NovaSeq 6000 platform (Illumina, San Diego, CA, USA) to produce 150 bp paired end reads, as previously described^18^. The Ribo-Zero^™^ Magnetic Kit (Human & Bacteria, Epicentre, Madison, WI, US) was used for ribosomal RNA depletion, and libraries were prepared using the NEBNext^®^ Ultra^™^ RNA Library Prep Kit (New England BioLabs Inc.^®^, Ipswich, MA, USA). Approximately 50 million reads (15 Gb raw data) were produced per sample on an Illumina NovaSeq 6000 instrument. Raw sequencing reads were deposited at the National Center for Biotechnology Information Sequence Read Archive (NCBI SRA) under BioProject ID PRJNA815861.

Raw sequencing data was parsed through the Metafunc pipeline^19^, which performs read preprocessing, host gene mapping and microbiome species identification. Further details of the computational pipeline may be found at https://gitlab.com/schmeierlab/workflows/metafunc, and complete analysis of this article is available at https://gitlab.com/alsulit08/uoc_response_rectalca. For the microbiome analysis part of the pipeline, no abundance filtering was performed at this stage of the analysis.

### Microbiome data preprocessing

For the microbiome data set, raw counts of microbe taxonomies were gathered into a Phyloseq object^20^, with metadata information on their response and tumour or normal status. Prefiltering of the species was then performed before the analysis, only including those within the Bacterial Kingdom, and those with at least 10 reads in 20 per cent of the samples (*Table S1*).

### Computational analyses and statistics

Expression levels for each human gene and sample were generated by the MetaFunc pipeline^19^, and differential human gene expression analysis (DGEA) using DESeq2^21^ was used to detect DEGs. To detect DEGs that were significantly differentially expressed in the tumour relative to each participant’s normal tissue between groups of responders, the model fitted by DESeq2 included covariates for response (complete or other), tissue type (tumour or normal), response:participant (index) and response:tissue. Care was taken to ensure the model matrix was of full rank, the model converged and that modelling assumptions were met. The genes were considered differentially expressed if their adjusted *P* values were <0.100.

From the results of this DESeq2 comparison, a preranked list of all resulting genes based on *P* values and log_2_-fold change was generated, and these genes were used as input for gene set enrichment analysis (GSEA) using clusterProfiler^22^ with the C5 Ontology Gene Sets collection (version 7) from the molecular signatures database (MSigDB)^23,24^. Specifically, the genes were ranked using the formula:


$$\mathrm{rank}=-\log\;10(P\;\mathrm{value})\;*\;\mathrm{sign}(\log_{2}\;\mathrm{FoldChange})$$

This ranking places the genes with lowest *P* values and positive log_2_-fold change at the top of the list, and the genes with lowest *P* values and negative log_2_-fold change at the bottom of the list. Genes at the top of the list contribute to gene sets with positive enrichment scores and genes at the bottom contribute to gene sets with negative enrichment scores. Gene sets were significantly enriched in a responder group if their adjusted *P* values were <0.050.

For the differential metatranscriptome analysis of the microbiome data set, the same model for group-specific condition effects (see DGEA above) to obtain differentially abundant (DA) microbes in tumour samples compared with matched normal samples, specific to complete responders compared with other responders, was used. Differentially abundant bacteria were considered those species with adjusted *P* values <0.100.

The correlation between DEGs and differentially abundant bacteria in rectal cancer was then investigated. Using the rlog transformed values for gene expression and microbial abundance obtained from DESeq2 of 87 identified DEGs and 10 DA bacterial species, a Spearman correlation analysis between each gene and species was performed, correcting the final *P* values using Benjamini–Hochberg (BH) adjustment. Influential points (species rlog > 12) were removed for the Spearman correlation calculation and corresponding scatter plots.

The α-diversity (microbial diversity within a sample or community) between different groups was compared using Observed (richness—number of observed taxa) and Shannon (richness and evenness—taking abundance of different taxa into consideration) measures. The sample set, as described in the microbiome preprocessing section above, was rarefied to 90 per cent of the smallest sample size in the data set and analysed in Phyloseq^20^. Observed and Shannon measures were calculated through the estimate_richness() function of the Phyloseq^20^ package, and compared between groups using Wilcoxon tests. Non-metric multidimensional scaling (NMDS) plots based on Bray–Curtis distances were used to visualize β-diversity between groups.

Analyses for DGEA, GSEA, DA, correlation and diversity were completed using R^25,26^ packages.

## Results

### Study population

This cohort comprised 40 patients (20 patients from each hospital) with diagnosed rectal cancer who were subsequently treated with CRT followed by surgical resection (*Table 1*). One patient was subsequently excluded, due to treatment cessation for palliative care. The majority (*n* = 36) were treated with LCCRT, with either capecitabine, FOLFIRI or 5FU. Two patients did not complete LCCRT due to the development of grade 3 toxicity. One patient received short-course radiotherapy, while the remaining two patients received sandwich CRT (FOLFOX). There were 12 females and 27 males, who ranged in age from 29 to 86 years (mean age: 62 years). There were five patients with complete response to LCCRT, five patients with near-complete response, 18 patients with incomplete response and eight patients who did not respond to LCCRT. In addition, three patients developed progressive disease or died of disease during the course of therapy, and these patients were also designated non-responders. Median follow-up was 42 months. Six patients died of the disease during a minimum follow-up period of 24 months.

**Table 1:** Characteristics of the patients with rectal cancer cohort

	All (*n* = 39)	Complete response (*n* = 5)	Near-complete response (*n* = 5)	Incomplete response (*n* = 18)	No response (*n* = 11)
**Age (years), mean ± SD**	62.0 ± 14.5	66.2 ± 9.3	61.2 ± 4.8	62.4 ± 14.8	69.9 ± 19.3
**Sex**					
Male	26 (66.7)	4 (80.0)	4 (80.0)	13 (72.2)	5 (45.5)
Female	13 (33.3)	1 (20.0)	1 (20.0)	5 (27.8)	6 (54.5)
**Clinical stage**					
1	1 (2.6)	0 (0.0)	1 (20)	0 (0.0)	0 (0.0)
2	4 (10.3)	0 (0.0)	0 (0.0)	2 (11.1)	2 (18.2)
3	25 (64.1)	4 (80.0)	4 (80)	11 (61.1)	6 (54.5)
4	9 (23.1)	1 (20.0)	0 (0.0)	5 (27.8)	3 (27.3)
**Death due to disease progression**	5 (12.8)	0 (0.0)	0 (0.0)	3 (16.7)	2 (18.2)
**Treatment**					
LCCRT (no other data)	4 (10.3)	0 (0.0)	0 (0.0)	3 (16.7)	1 (9.1)
LCCRT (capecitabine)	26 (66.7)	5 (100.0)	3 (60.0)	9 (50.0)	9 (81.8)
LCCRT (5FU)	3 (7.7)	0 (0.0)	0 (0.0)	3 (16.7)	0 (0.0)
LCCRT (FOLFIRI)	2 (5.1)	0 (0.0)	0 (0.0)	2 (11.1)	0 (0.0)
Sandwich CRT	2 (5.1)	0 (0.0)	2 (40.0)	0 (0.0)	0 (0.0)
SCRT	1 (2.6)	0 (0.0)	0 (0.0)	1 (5.6)	0 (0.0)
Palliative	1 (2.6)	0 (0.0)	0 (0.0)	0 (0.0)	1 (9.1)

Values are *n*(%) unless otherwise stated. LCCRT, long-course chemoradiotherapy; FOLFIRI, folinic acid, fluorouracil and irinotecan; SCRT, short-course radiotherapy; 5FU, fluorouracil.

### Differential gene expression and gene set enrichment between response groups

**Fig. 1:**
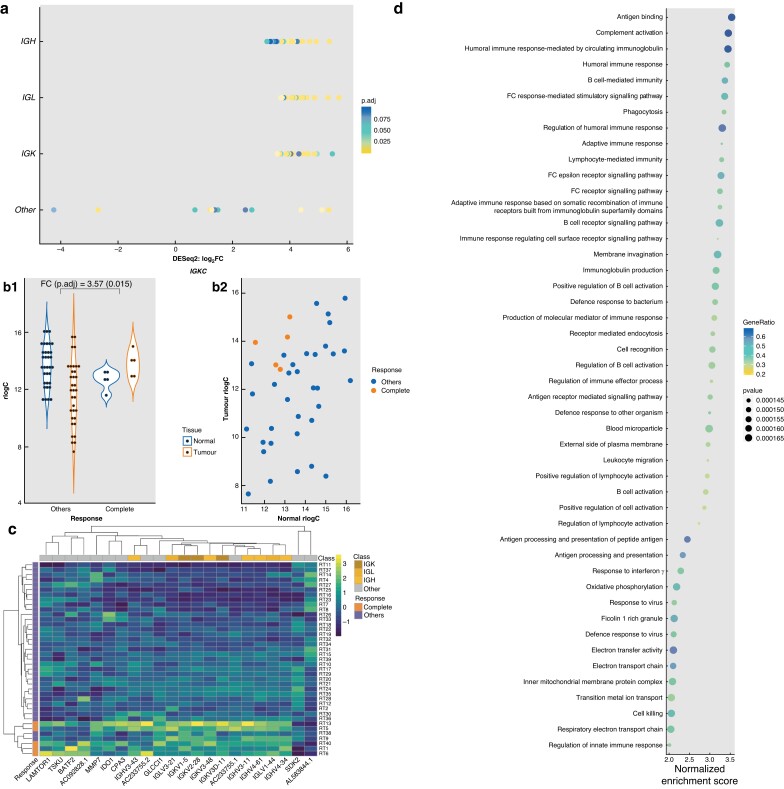
Host differential gene expression and gene set enrichment analysis **a** Differentially expressed genes (DEGs) classified into immunoglobulin (Ig)-related genes and other non-Ig genes. *p.adj,* adjusted *P* value. **b** Transformed counts (rlog) of Tumour/Normal for complete responders compared with other responders of a representative DEG, *IGKC*. **b1** Tumour *versus* Normal for each response category (FC, log_2_-fold change*; p.adj*, adjusted *P* value). **b2** Tumour values and their corresponding normal values per sample by response. **c** Heatmap of the top 10 (by *adjusted P* values) DEGs, which are all Ig-related genes, and the other 12 non-Ig DEGs clustered by sample and gene. **d** Top 47 positively enriched gene sets (*y* axis) in Tumour *versus* Normal of Complete Responders. All have adjusted *P* values (Benjamini–Hochberg) of 0.00928. Gene ratio is the ratio of core enrichment genes to the gene set size. Gene sets coloured yellow are immune-related gene sets. Gene sets coloured red are gene sets that refer to a response to microbial input. All other gene sets are coloured black.

DEGs were analysed between matched pairs of tumour and normal tissues, to account for interpersonal variation in gene expression, and then these DEGs were compared between response groups. Eighty-seven genes were found to be differentially expressed between tumour and normal samples (adjusted *P* value <0.100), and only seen in complete responders. Interestingly, the majority of these genes are associated with immunoglobulin chains, with 75 of 87 genes having prefixes of *IGH-*, *IGL-* or *IGK-* (*Fig. 1a*), for immunoglobulin heavy, light, and kappa respectively. Tumour *versus* normal rlog was plotted as transformed counts per sample of representatives from these genes and showed that for most of these genes, complete responders cluster at high tumour–low normal values (upper left quadrant), indicating that in the complete responder group, these genes are more highly expressed in tumours compared with normal tissues (*Fig. 1b* and *Fig. S1*). When DGEA was carried out comparing non-responders to all other responders, no DEGs were identified between the two groups.

The majority of significant DEGs were upregulated in tumours compared with normal tissue. The top 10 DEGs (all immunoglobulin related), in addition to 12 non-immunoglobulin-related DEGs, could robustly distinguish complete responders from all other patients, as illustrated in *Fig. 1c* and *Fig. S2.*

Subsequently, Gene Set Enrichment Analysis (GSEA), which ascribes function to DEGs, was performed using genes ranked according to DESeq2 *P* value and log_2_-fold change (±). Gene sets relating to immune responses constitute the majority of the top 47 enriched gene sets in tumours of complete responders (*Fig. 1d*). Among the gene sets with the highest enrichment scores were Complement Activation and B Cell Mediated Immunity, consistent with the majority of the 87 DEGs in complete responders being related to immunoglobulins.

### Diversity analysis of the microbiome of rectal tumours

No significant differences were found in Observed or Shannon measures of α-diversity (*Fig. S3A–H*). Any distinct group clustering by NMDS was not detected, indicating similar β-diversity between the response groups (*Fig. S3I–L*).

### Differential abundance analysis of bacterial species between radiotherapy response groups

**Fig. 2:**
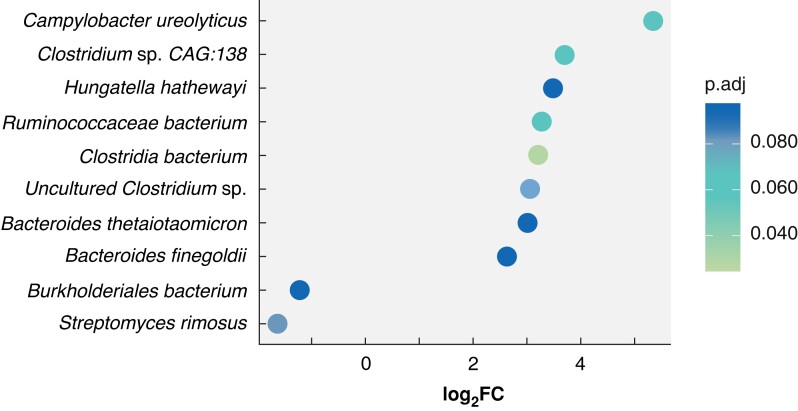
Differentially abundant bacteria in tumour samples compared with matched normal tissue, specific to complete responders Plot of the 10 differentially abundant bacteria showing their log_2_-fold changes (*x* axis) and adjusted *P* (p.adj) values by colour.

A similar approach to DGEA in the human data set was used to identify bacterial taxa that were differentially abundant in tumour *versus* matched normal samples, and specific to complete responders. Analysis of differences in the tumour microbiome between response groups identified 10 bacterial species that were differentially abundant (adjusted *P* values t <0.100) in tumour tissue compared with matched normal tissue in complete responders (*Fig. 2*). Bacterial species identified included *Ruminococcaceae bacterium*, *Hungatella hathewayi*, *Bacteroides thetaiotaomicron* and *Clostridium* species^27–32^. Plotting tumour *versus* normal rlog transformed counts per sample of these microbes showed that there is a separation between the complete responders compared with other responders, although not as pronounced as that seen in human DEGs (*Fig. S4*).

### Correlation between host gene expression and microbial abundances

**Fig. 3:**
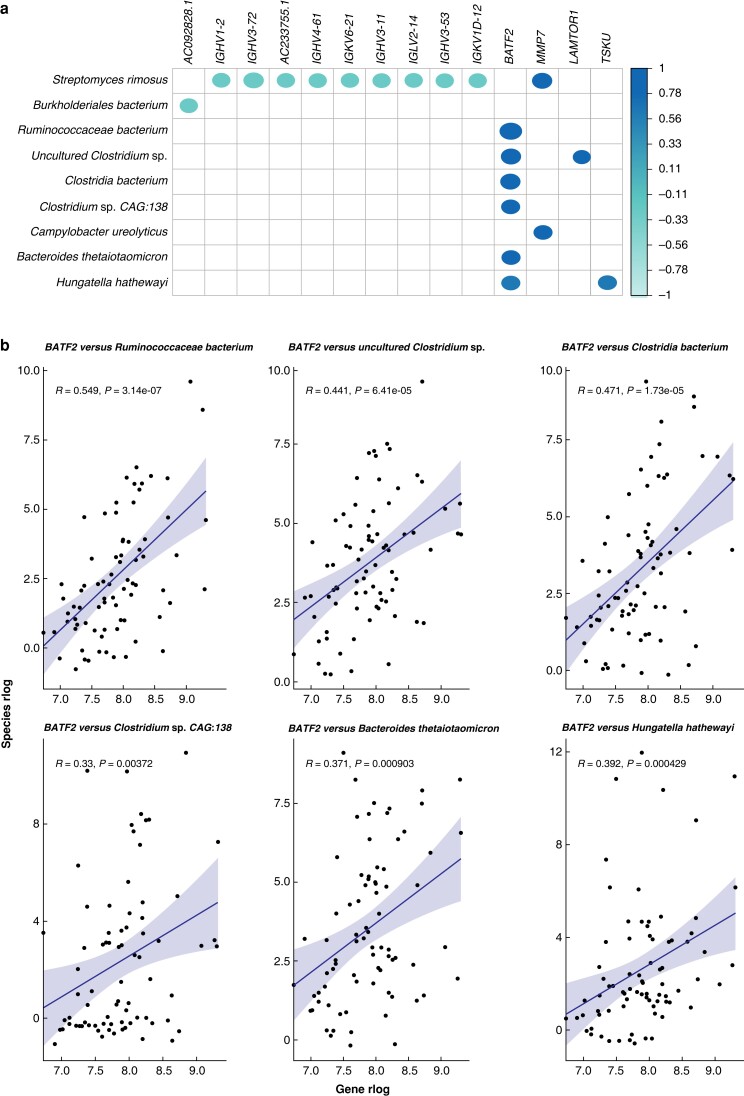
Correlations between differentially expressed genes and differentially abundant microbes **a** Correlation plot showing bacteria–gene correlations with at least one significant correlation (adjusted *P* value *<0.1*00). Spearman correlation coefficients (ρ) are represented by the colour bar; blank spaces represent correlations that are not significant. **b***BATF2* and its positive correlations with differentially abundant bacteria. R, Spearman coefficient; e, base number x10^power^.

Significant correlations between DEGs and differentially abundant bacterial species were identified. Among DEGs that positively correlated with the microbial abundances, the *BATF2* gene was notable. Positive correlations (Spearman coefficient: 0.355–0.549; BH-adjusted *P* value: 1.661 × 10^−4^−7.699 × 10^−2^) of this gene with several DA bacterial species, previously linked to CRC, including *Ruminococacceae bacterium* and *Bacteroides thetaiotaomicron* (*Fig. 3*), were found.

## Discussion

The identification of predictive biomarkers of response to CRT will result in improved survival, a reduction of morbidities rate related to unnecessary treatment, and a more targeted approach to treatment for patients with rectal cancer. Previous studies have attempted to identify markers of complete response to CRT, and while clinicopathological and radiological features have been identified, they are limited in sensitivity and specificity^1^. Intratumoural heterogeneity contributes to a lack of reproducibility between molecular biomarker studies, and as a result, no biomarker is currently in clinical use^1,8^. In order to identify a predictive biomarker of response for rectal tumours, a mechanistic link to the underlying tumour biology is also warranted. This study identifies potential biomarkers of complete response to CRT in patients with rectal cancer, in addition to uncovering novel links between the tumour microbiome and immune response in the rectal tumour microenvironment. Tumour tissues were firstly compared with their corresponding matched normal tissues, to mitigate interpersonal differences that are not due to tumour characteristics.

Rather than a continuum of gene expression changes from complete responders to non-responders, a tight clustering of complete responders based on gene expression of mainly immune-related genes, compared with all other patients, was observed. This suggests the presence of a distinct tumour microenvironment in a group of patients that predisposes them to a complete response to radiotherapy. In these cohorts, the patients showed significantly higher expression of genes responsible for complement activation and B cell-related functions in their tumour tissue compared with adjacent normal tissue, supporting previously published reports that immunoglobulins may recognize radiotherapy-induced neoantigens resulting in complement activation and CD8+ T cell responses^33^.

Enriched gene sets related to antigen presentation in the cohort of complete responders were also observed. Ionizing radiation can cause DNA mutations, which, when translated into peptides, are presented on MHC-1 molecules, eliciting a cytotoxic immune response. This has been observed in studies of other cancer types, where radiation has been shown to recruit neutrophils and monocytes, as well as promote maturation of antigen presenting cells^34^. Radiotherapy also results in mimicry of viral infections, where cytosolic DNA induces the release of type 1 interferons that recruit dendritic cells specialized in antigen presentation to CD8+ T cells^35^. Our findings indicate that enhanced antigen presentation in pretreatment tumours predisposes them to a successful response to CRT.

A significant enrichment of the *BATF2* gene was found in patients who went on to have a complete response to CRT, which is consistent with findings that *BATF2* depletion in tumour compared with normal tissue is correlated with poor prognosis in CRC^36^. *BATF2* is thought to be induced by type 1 interferons and play a role in viral infections^37^ and may, as a result, contribute to radiotherapy-induced responses that mimic viral infection. Type-1 interferons are important players in antiviral immune responses, recruiting dendritic cells specialized in antigen presentation to CD8+ T cells^35^. This is again consistent with viral infection and antigen presentation-enriched gene sets identified in complete responders in this cohort. Furthermore, *BATF2* may also induce antitumour effects through the induction of CD8^+^ T cells^38^.

The enrichment of immune response-related genes and gene sets in tumours of complete responders to CRT in our cohort is consistent with the findings of a previous study^39^. In that study, they identified antigen presentation, interferon (IFN) activity and B cell activity to be enriched in good responders to preoperative CRT in locally advanced rectal cancer (LARC) patients, and that these factors may relate to good responses to the treatment via activation of an antiviral-like response and CD8+ T cell recruitment. While their study largely used microarray gene expression and targeted sequencing of CRC genes, this study mainly used whole RNA sequencing, which also allowed investigation of possible contributions of the microbiome.

A key finding of this study is the enrichment of gene sets relating to response to bacteria in complete responders. The role of the microbiome in therapy response has recently become a focal point for studies of different types of treatments in a variety of cancer types. Early studies of the microbiome in CRC had suggested a decrease in microbial diversity associated with tumours compared with healthy controls^10,11,40^. However, more recent studies refute this finding, where CRC samples have increased richness^41^ or are no different^27^ compared with controls. Consistent with these more recent studies^42^, here, no difference was found in microbiome diversity metrics between the response groups. However, a number of bacterial species that were differentially abundant in tumours of complete responders were identified, several of which had previously been implicated in CRC carcinogenesis and prognosis. *Hungatella hathewayi* has been reported to be differentially abundant in CRC^27^, and has been reported to drive methylation of tumour suppressor genes^28^ and was also found to be significantly more abundant in complete responders in the present study. *Ruminococcaceae*, a putative commensal genus, has been linked to the expression of T cell recruiting chemokines^31^, indicating a role in tumour-killing immune activation, and a low abundance has been associated with CRC^29,30^. High levels of this genus may indicate a beneficial role in driving response to CRT in the tumours of complete responders through enhanced T cell activation. Similarly, *Clostridium* species have been associated with a protective effect against CRC, due to their ability to synthesize short-chain fatty acids, notably butyrate, which can promote CRC cell apoptosis and inhibit carcinogenesis^32^.

The importance of the gut microbiome in response to immunotherapy has garnered much attention in recent years, where species of microbes have been associated with good response to immunotherapy treatments^13,14^. Given the similarities with the known immune mechanisms at play in radiotherapy response, for example CD8+ T cell activation, an overlap between the established systemic effect of the microbiome on immunotherapy efficacy and that of radiotherapy is likely. *Ruminococcaceae* and *Bacteroides thetaiotaomicron*, both enriched in complete responders in our cohort, have been reported to enhance immunotherapy effects^13,43^, and this mechanism may also contribute to radiotherapy-induced immune clearance of cancer cells. Indeed, a previous study^13^ showed that there is a correlation between CD8+ T cells and abundance of the *Ruminococcaceae* family, and that there are statistically more CD8+ T cells in responders *versus* non-responders to immunotherapy in melanoma patients. Taken together with the associations between differentially abundant bacteria and *BATF2* expression, and the increased abundance of commensal bacteria, such as *Clostridium* spp., the activation of immune pathways in the tumour microenvironment indicates a potential role of the tumour microbiota in the radiotherapy response in rectal cancer. These findings also suggest that there may be value in changing the gut microenvironment (for example through faecal microbiota transplants) as a means of improving the efficacy of CRT, in a similar manner to that reported for immunotherapy. Nevertheless, future studies to assess the clinical utility of incorporating immune and bacterial gene markers to stratify patients for targeted therapy are necessary in larger prospective cohorts.

This study has some limitations: one is the possible selection bias this study has in collecting from two different centres (Christchurch Hospital in New Zealand and the Peter MacCallum Cancer Centre in Melbourne), although a mixture of responses come from both study groups. In addition, this study is computational and further laboratory testing of identified genes, microbes and mechanisms in experimental procedures is highly advocated.

Finally, the results reported here suggest that an increase in the expression of genes contributing to immune activation in tumours compared with normal samples contributes to radiosensitivity. The main hypothesis was that this primes a microenvironment that activates antitumour responses during radiotherapy. Furthermore, bacteria enriched in the tumours of complete responders in this study have previously been associated with CRC and improved efficacy of immunotherapy, and could possibly contribute to the immune activation taking place in complete response to radiotherapy. These data provide future targets for biomarker validation and provide direction to investigate the mechanisms of radiotherapy response in rectal cancer.

## Supplementary Material

zrad035_Supplementary_Data

## Data Availability

Sequencing data can be found under Bioproject ID PRJNA815861 in the NCBI SRA database. Details for bioinformatics and statistical analyses may be found at https://gitlab.com/alsulit08/uoc_response_rectalca. Limited patient metadata may be provided upon reasonable request to the authors.
