# Supplementary material for: Human gene and microbial analyses in rectal cancer complete responses to radiotherapy
Source: BJS Open. 2023 May 10;7(3):zrad035. doi: 10.1093/bjsopen/zrad035 (PMC10170257; doi:10.1093/bjsopen/zrad035)
Supplement: zrad035_Supplementary_Data [file zrad035_supplementary_data.docx]

Human gene and microbial analyses in rectal cancer complete responses to radiotherapy

Arielle Kae Sulit^1,2,*^, Kasmira Wilson^4,5,7,*^, John Pearson^3^, Olin K. Silander^1^, Shienny Sampurno^5^, Michael Michael^6,7^, Robert Ramsay ^4,5^, Alexander Heriot^4,7^, Frank Frizelle^2^, Rachel Violet Purcell^2^

^1^School of Natural Sciences, Massey University, Auckland, New Zealand

^2^Department of Surgery, University of Otago, Christchurch, New Zealand

^3^Biostatistics and Computational Biology Unit, University of Otago, Christchurch, NZ

^4^Department of Surgical Oncology, Peter MacCallum Cancer Centre, Melbourne, Australia

^5^Differentiation and Transcription Laboratory, Sir Peter MacCallum Cancer Centre, Melbourne, Australia

^6^Department of Medical Oncology, Peter MacCallum Cancer Centre, Melbourne, Australia

^7^Sir Peter MacCallum Department of Oncology, University of Melbourne, Melbourne, Australia

^*^Authors have contributed equally

**Corresponding author.** Arielle Kae Sulit; University of Otago, Christchurch; *ORCID:* 0000-0002-3643-3348

**Supplementary Materials - Index**

| **Supplementary Figures and Tables** |  |
| --- | --- |
| Table S1 | *pag. 2* |
| Figure S1 | *pag. 3* |
| Figure S2 | *pag. 4* |
| Figure S3 | *pag. 5* |
| Figure S4 | *pag. 6* |
|  |  |
|  |  |
|  |  |
|  |  |
|  |  |
|  |  |
|  |  |

**Supplementary Figures and Tables**

| **Table S1. Summary statistics of sample read counts at each stage of filters applied** | | | |
| --- | --- | --- | --- |
|  | **All Species** | **Bacterial Species** | **Bacterial species (≥10 reads in ≥ 20% samples)** |
| **Minimum** | 216192 | 196204 | 184911 |
| **Maximum** | 5931870 | 5882950 | 5845561 |
| **Mean** | 720689.8 | 676079.1 | 652712.7 |
| **Median** | 519818.5 | 481973 | 468629 |

| 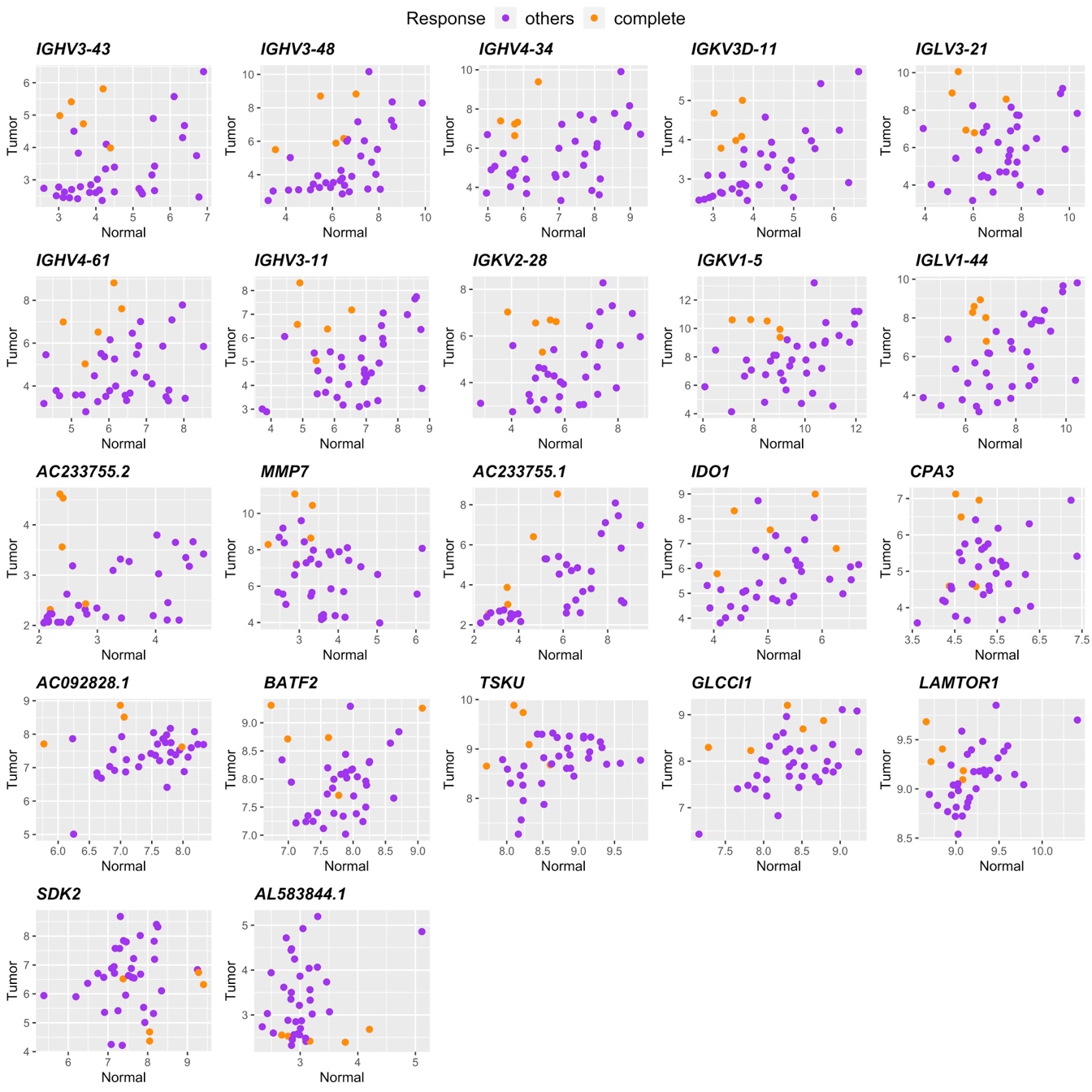 |
| --- |
| **Figure S1. *rlog* values of the** **differentially expressed genes in tumor samples of complete responders, compared to their corresponding matched normal samples.** We see that for many of these genes, complete responders (orange) cluster at the upper-left quadrant of the plots distinct from other responders (purple) except for *SDK2*, and *AL583844.1* which have negative log_2_ fold changes. |

| 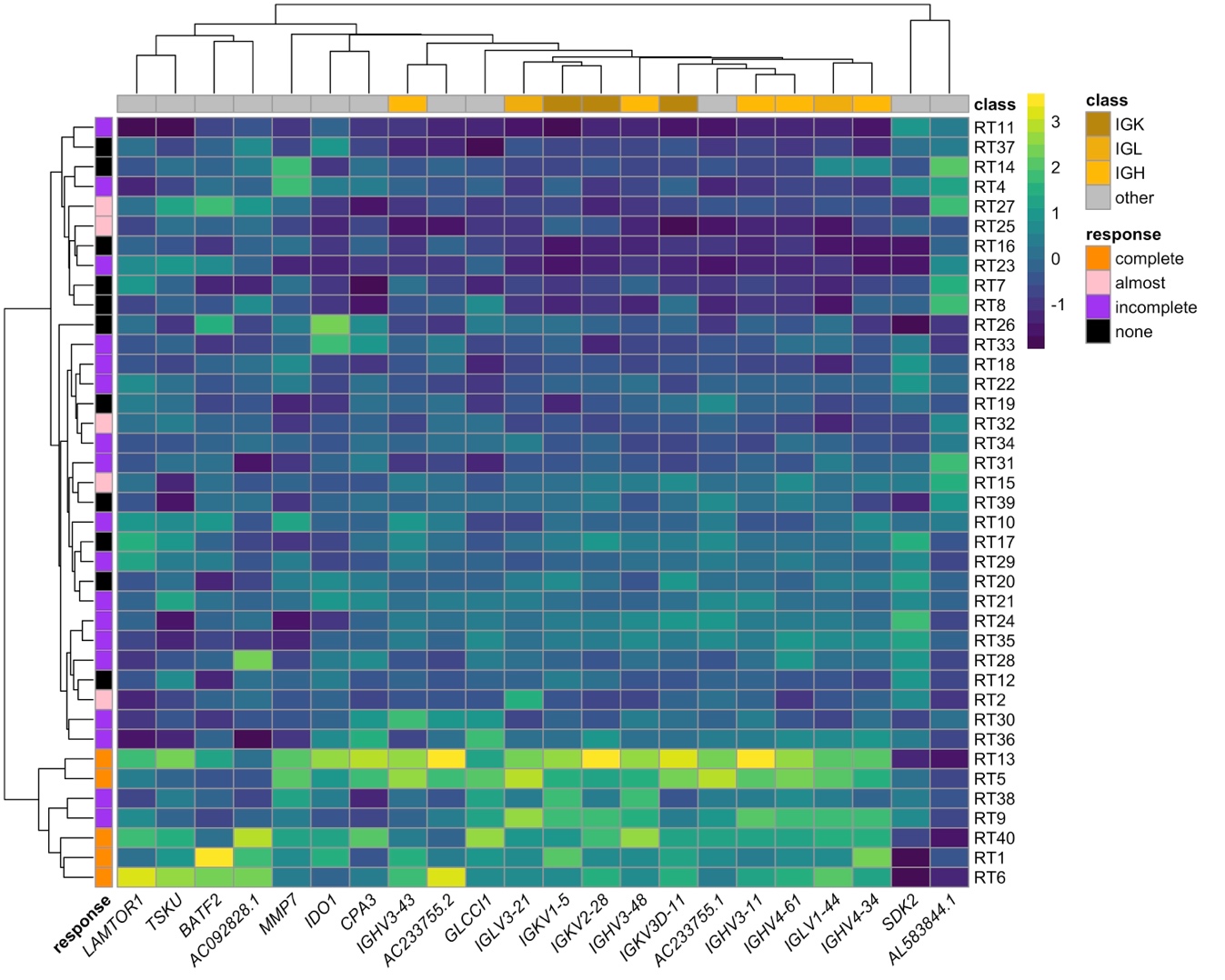 |
| --- |
| **Figure S2. The top 10 DEGs (all Ig-related genes), and the other 12 non-Ig DEGs separate complete responders from other response groups.** Genes were ranked by adjusted p-values. We obtained the tumor vs normal (T/N) ratios of these genes using rlog transformed counts per patient sample, and scaled these values as z-scores per gene (heatmap colors). Heatmap and clusters were generated by the *pheatmap*^1^ function and library in R. |

| 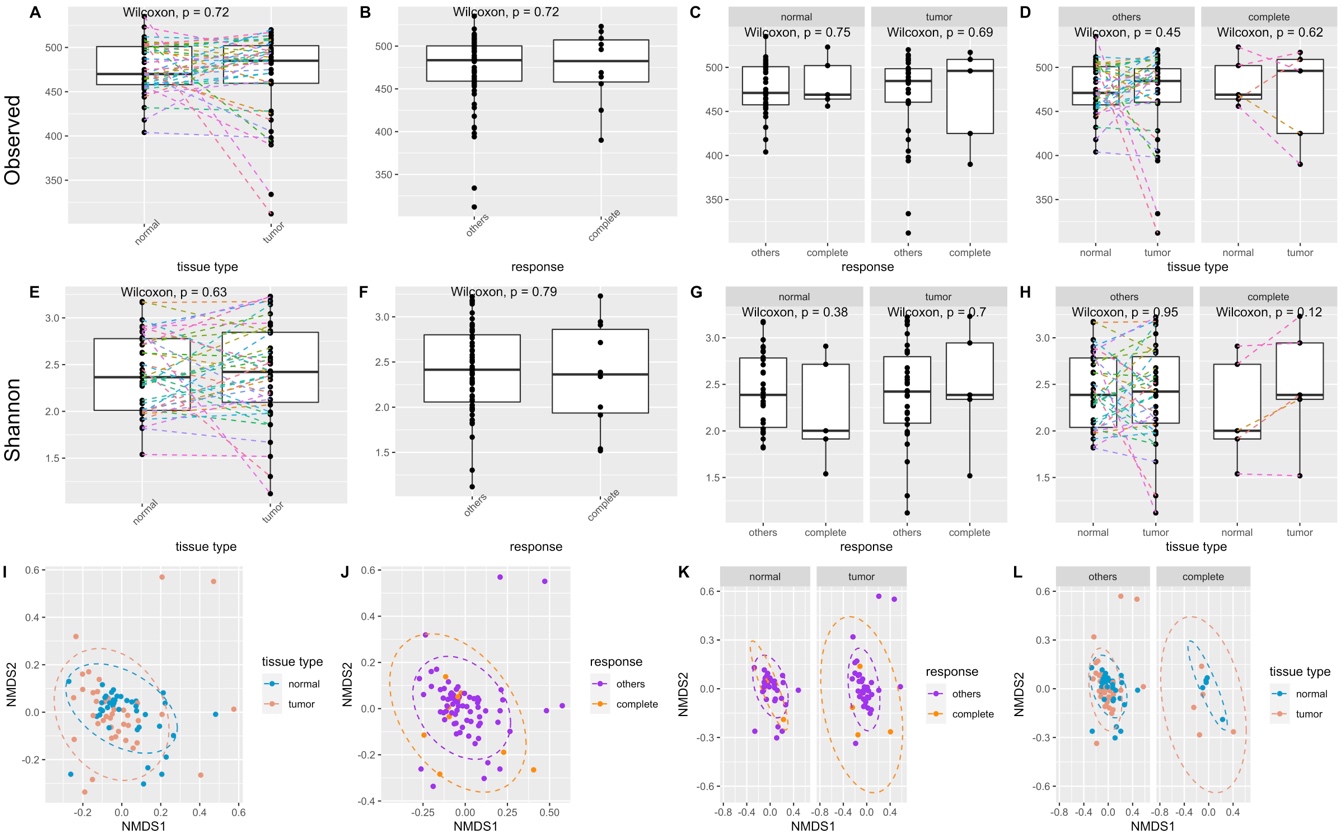 |
| --- |
| **Figure S3. Diversity analyses on different grouping combinations of tumor, normal, and response groups in rectal cancer. A-D:** Observed Alpha Diversity. **A.** between normal and tumor, **B.** between response groups, **C.** between response groups within tissue type, **D.** between tissue type within response groups. **E-H**. Shannon Alpha Diversity. **E.** between normal and tumor, **F.** between response groups, **G.** between response groups within tissue type, **H.** between tissue type within response groups. **I-L**. NMDS ordination of Bray-Curtis distances between samples, grouped by: **I.** between normal and tumors, **J.** between response groups, **K.** between response groups within tissue types, **L.** between tissue type within response groups. |

| 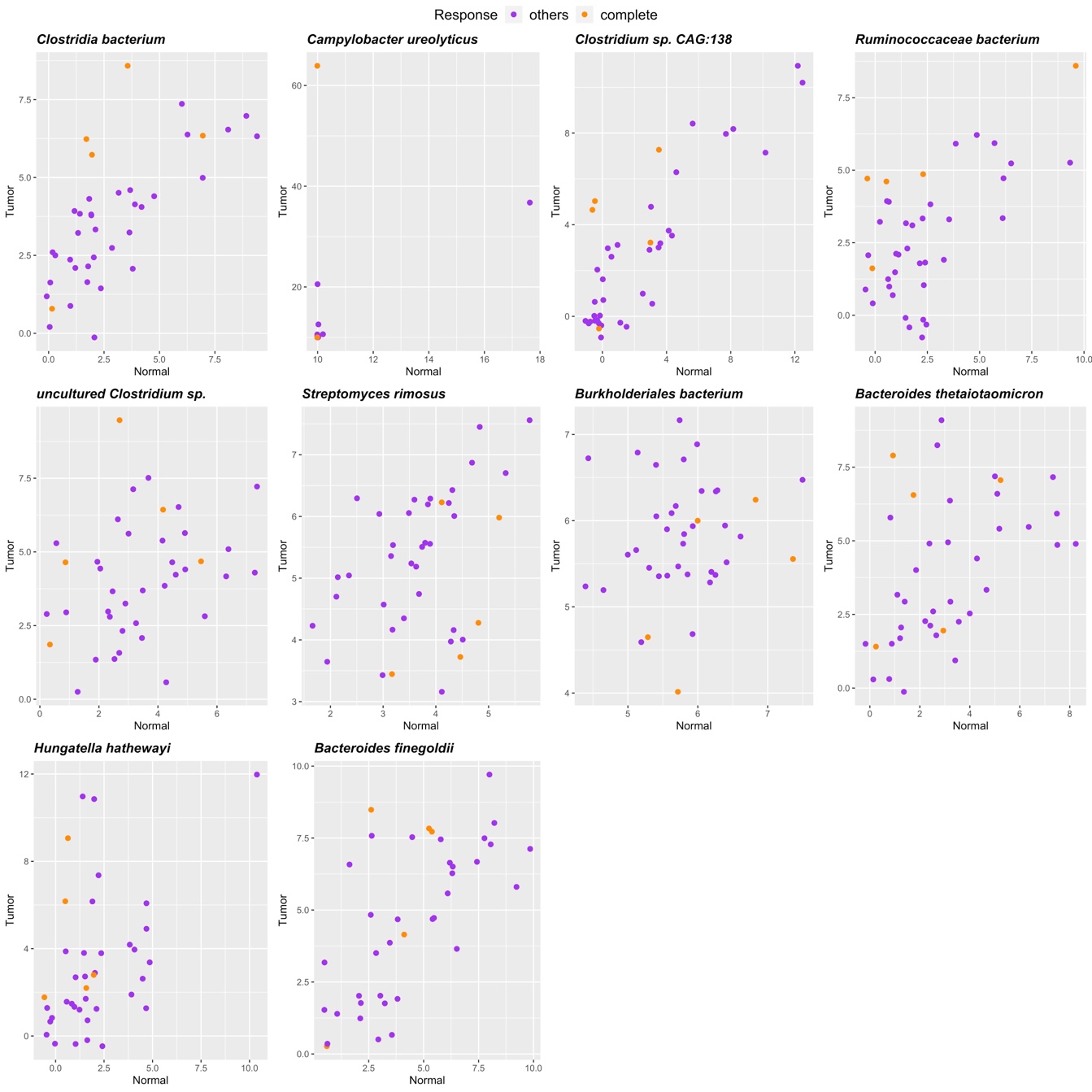 |
| --- |
| **Figure S4. Differentially abundant bacteria in tumor samples compared to matched normal tissue, specific to complete responders**. Scatterplot between rlog values of the bacterial species in tumor samples and their corresponding matched normal samples. |

**Reference:**

1. Kolde, R. pheatmap: Pretty Heatmaps. (2019).
